# Intracellular Signaling Transduction Pathways Triggered by a Well-Known Anti-GHR Monoclonal Antibody, Mab263, *in Vitro* and *in Vivo*

**DOI:** 10.3390/ijms151120538

**Published:** 2014-11-10

**Authors:** Hainan Lan, Wei Li, Hailong Jiang, Yanhong Yang, Xin Zheng

**Affiliations:** 1 College of Animal Science and Technology, Jilin Agricultural University, Xincheng Street 2888, Changchun 130118, China; E-Mails: lan2012172@163.com (H.L.); lanbotonglan@163.com (H.J.); lilantougao@163.com (Y.Y.); 2 State Key Laboratory of Animal Nutrition, China Agricultural University, Beijing 100193, China; E-Mail: weiv_li@hotmail.com

**Keywords:** monoclonal antibody 263 (Mab263), growth hormone receptor, signal transduction pathway

## Abstract

A series of studies have reported that monoclonal antibody 263 (Mab263), a monoclonal antibody against the growth hormone receptor (GHR), acts as an agonist *in vitro* and *in vivo*. However, the intracellular signaling pathways triggered by Mab263 have not yet been delineated. Therefore, we examined the intracellular signaling pathways induced by Mab263 *in vivo* and *in vitro* in the present study. The results show that this antibody activated janus kinase 2 (JAK2), signal transducer and activator of transcription 3 (STAT3), STAT1 and extracellular signal-regulated kinase 1/2 (ERK1/2), but not STAT5. The phosphorylation kinetics of JAK2, STAT3/1 and ERK1/2 induced by Mab263 were subsequently analyzed in dose-response and time course experiments. Our observations indicate that Mab263 induced different intracellular signaling pathways than GH, which indicates that Mab263 is a signal-specific molecule and that Mab263 may be a valuable biological reagent to study the mechanism(s) of GHR-mediated intracellular signaling pathways.

## 1. Introduction

Growth hormone (GH) is a 22-kDa, 191-amino acid peptide that is secreted by the anterior pituitary and has anabolic, pro-proliferative, anti-apoptotic and metabolic effects in various target tissues [1]. GH is an asymmetric molecule, and its interaction with growth hormone receptor (GHR) is mediated by two non-equivalent binding sites (site 1 and site 2) [2]. The binding of GH to its specific receptor (GHR) on the surface of target cells induces receptor conformation change(s) to activate the intrinsic tyrosine kinase, janus kinase 2 (JAK2), and initiates intracellular signaling cascades [3]. These events lead to the tyrosine phosphorylation of multiple cellular proteins, including signal transducers and activators of transcription (STAT) (STATs 1/3/5) and extracellular regulated kinases (ERK1/2). These signaling pathways act together to contribute to the overall actions of GH [3].

Anti-GHR monoclonal antibodies have been an important tool for exploring the mechanisms of GHR activation. Monoclonal antibody 263 (Mab263) (commercially available) is a well-known and documented anti-GHR antibody that was initially developed by Barnard *et al.* [4], and a series of intensive studies relevant to Mab263 agonistic activity have been reported over the past decades [5,6,7,8,9,10]. The crystallographic data of the GH-(GHR)_2_ complex have suggested that one GH molecule dimerizes two GHR molecules, which is followed by the initiation of signaling events [2,8]. Subsequently, Fuh *et al.* [8] initially found that a bivalent anti-GHR monoclonal antibody (Mab263) can activate a chimeric GHR in a proliferation assay; these findings have led to a model of hormone-induced sequential receptor dimerization. However, Rowlinson *et al.* [7] prepared a panel of anti-GHR Mabs and found that only one (Mab263) of 14 Mabs could activate the full-length GHR, which indicated that dimerization itself is not sufficient to activate the receptor, and a GHR conformational change is likely required for GHR activation. Carlsson *et al.* then reported that Mab263 could promote the growth of hypophysectomized rats, although it did not exhibit the insulin-like actions of GH, which require STAT5 activation [5]. Furthermore, Mab263 caused a concentration-dependent stimulation of fatty acid oxidation, an effect similar to GH [10]. The majority of Mab263 epitope residues are discontinuously distributed on the β-turn loop at residues 79–96 and on the loops between the β-strands of subdomain 1 of GHR ECD based on an epitope map for Mab263 [9]. The Mab263 induces similar, but not identical, conformation changes as GH by a modelling analysis [9]. Mab263 has been used based on its agonistic properties; however, the intracellular signaling pathway(s) induced by Mab263 are unknown, even though this antibody has been extensively studied for its agonist property *in vitro* and *in vivo* [5,6,7,8,9,10].

In the present study, CHO (Chinese hamster ovary) cells transfected with rat GHR and 3T3-F442A cells expressing endogenous mouse GHR were used as cell models *in vitro* to investigate the intracellular signaling pathways induced by Mab263. In addition, we also investigated the intracellular signaling pathway induced by Mab263 *in vivo*. We found that Mab263 activates different intracellular signal transduction pathways, which may explain why Mab263 is a weak agonist compared to GH. The present study also suggests that Mab263 may be a valuable biological reagent to study the mechanism(s) of GHR-mediated intracellular signaling pathways. Moreover, these findings suggest that signal-specific molecules capable of introducing different biochemical responses can be produced.

## 2. Results and Discussion

### 2.1. Mab263 Binds to rGHR or mGHR Expressed on CHO-GHR638 or 3T3-F442A Cells

Numerous reports have indicated that Mab263 could bind to rat GHR [4,5,9], and Mab263 has been demonstrated to reorganize rat GHR expressed on CHO-GHR638 cells that were prepared and characterized in our lab [11].

**Figure 1:**
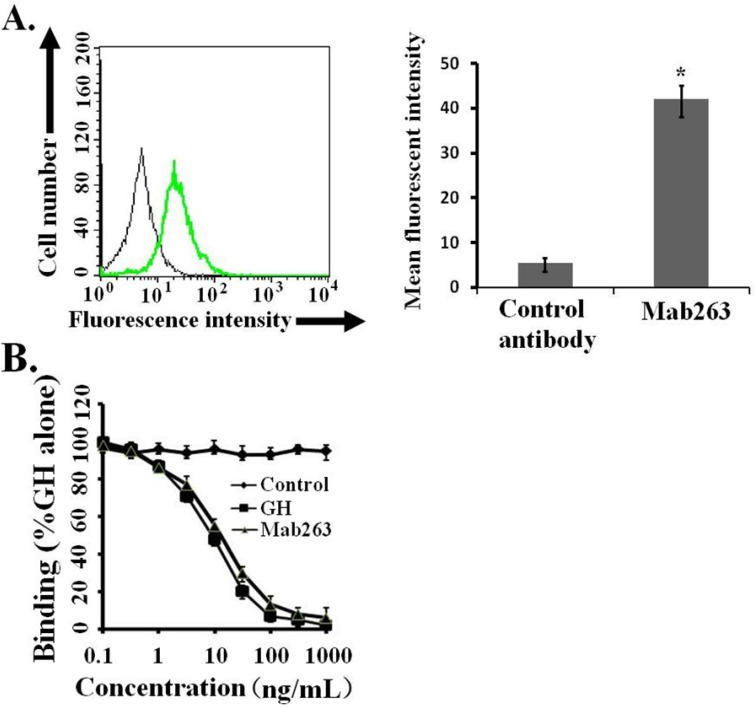
Monoclonal antibody 263 (Mab263) specifically binds to mouse growth hormone receptor (mGHR) expressed on the mouse GHR cell model. (**A**) Binding of fluorescein isothiocyanate-Mab263 (FITC-Mab263) to 3T3-F442A cells. The cells were pre-treated as described in the Materials and Methods. The cells were then incubated with FITC-Mab263 (green line, MFI (mean fluorescence intensity): 41.96) or FITC-control antibody (black line, MFI: 5.60) for 1 h in the dark at 4 °C. The cells were then washed, resuspended in 0.5 mL fluorescence-activated cell sorting (FACS) buffer and analyzed using a FACS Calibur Flow Cytometer. (**Right**) The corresponding histogram of the data of three separate FACS analyses. Data are shown as the mean ± SE. * *p* < 0.05 compared with the control value; and (**B**) Mab263 competes with GH for binding to GHR. The 3T3-F442A cells were pre-treated as described in the Materials and Methods. The cells were incubated with the indicated amounts of FITC-GH in the absence or presence of various concentrations of unlabeled GH, Mab263 or control antibody. After washing, the cells were resuspended in 0.5 mL FACS buffer and analyzed by flow cytometry. The data shown are the mean ± SE from three independent experiments.

Mouse 3T3-F442A pre-adipocytes, which were initially developed by Green [12] and endogenously express mouse GHR, have been demonstrated as a valuable system for studying the mechanism of action of GH and signaling pathways induced by GH [13,14,15,16]. In addition, these cells only express GHR and not PRLR (prolactin receptor) [17,18]. Mab263 could reportedly bind mouse GHR [19], although it shows poor immunoreactivity with the mouse GH receptor [9]. The ability of Mab263 to bind to GHR expressed on 3T3-F442A cells under our experimental conditions was assessed by flow cytometry. The 3T3-F442A cells treated with Mab263 showed cellular staining, and the difference between the control antibody-treated group and the Mab263-treated group was statistically significant (*p* < 0.05) (Figure 1A). In addition, a competitive receptor-binding assay was carried out to further confirm whether Mab263 specifically binds to the mGHR expressed on 3T3-F442A, and it also showed that unlabeled hGH displaced the fluorescein isothiocyanate-hGH (FITC-hGH) from cells, as expected (Figure 1B). Mab263 also displaced FITC-hGH in a dose-dependent manner. These results demonstrated that Mab263 binds to the mGHR expressed on 3T3-F442A under our experimental conditions.

### 2.2. Signaling Transduction Activated by Mab263 in CHO-GHR638 Cells

**Figure 2:**
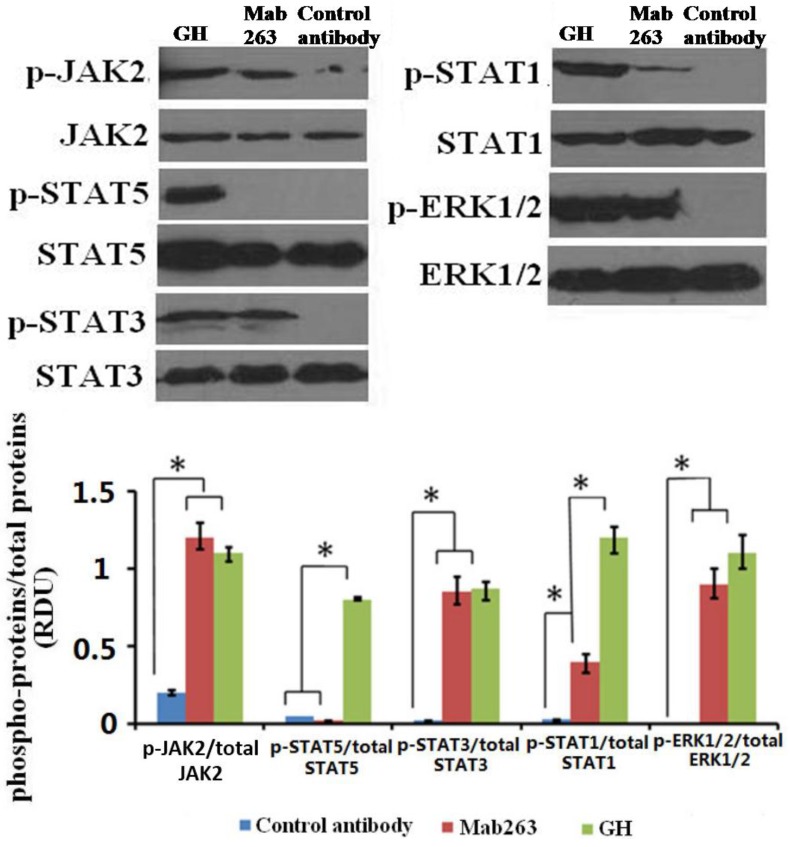
The intracellular signaling pathway(s) induced by Mab263 in the rat GHR cell model. CHO (Chinese hamster ovary)-GHR638 cells were pre-treated as described in the Materials and Methods. The CHO-GHR638 cells were challenged with GH (20 nM), Mab263 (20 nM) or control antibody (20 nM) for 30 min. The cells were solubilized in lysis buffer on ice for 30 min, and the cell extracts were then harvested and concentrated by ultrafiltration. The sample was separated on an SDS-PAGE gel and transferred to PVDF membranes. After blocking and washing, the membranes were probed with antibodies recognizing phosphorylation of janus kinase 2 (p-JAK2), phosphorylation of signal transducer and activator of transcription 1/3/5 (p-STAT1/3/5) and phosphorylation of extracellular signal-regulated kinase 1/2 (p-ERK1/2). After washing, secondary antibody was added to detect bound antibody using the ECL detection system. After detection, the membranes were stripped and re-probed with antibodies against the total JAK2, STAT1/3/5 and ERK1/2 proteins to verify equal protein loading in each lane; and (**Bottom**) The corresponding histograms of data from three separate western blot analyses. Densitometry data for p-JAK2, p-STAT1/3/5 and p-ERK1/2 were normalized to that of total JAK2, total STAT1/3/5 and total ERK1/2, presented as relative densitometry units (RDU) and expressed as the means ± SE. * *p* < 0.05 compared with the control values; p, phosphorylation.

We first detected the intracellular signaling molecule protein(s) activated by Mab263 in CHO-GHR638 by western blot analysis. CHO-GHR638 cells were treated with 20 nM of GH, Mab263 or a control antibody for 30 min and subsequently treated as described in the Materials and Methods. As illustrated in Figure 2, GH strongly activated JAK2, STAT1/3/5 and ERK1/2 in CHO-GHR638 cells, and Mab263 also induced the rapid phosphorylation of JAK2, ERK1/2 and STAT1/3, but not STAT5; the differences between the control antibody and the Mab263 treatments were statistically significant (*p* < 0.05). In addition, STAT1 is also activated by Mab263, but the level of phosphorylation is very weak compared to that of GH.

**Figure 3:**
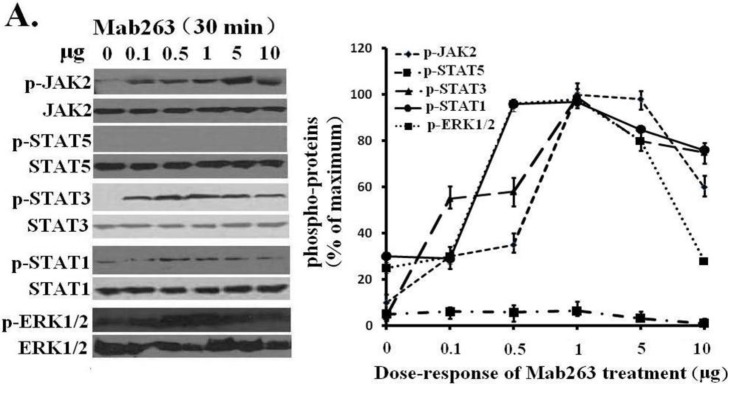

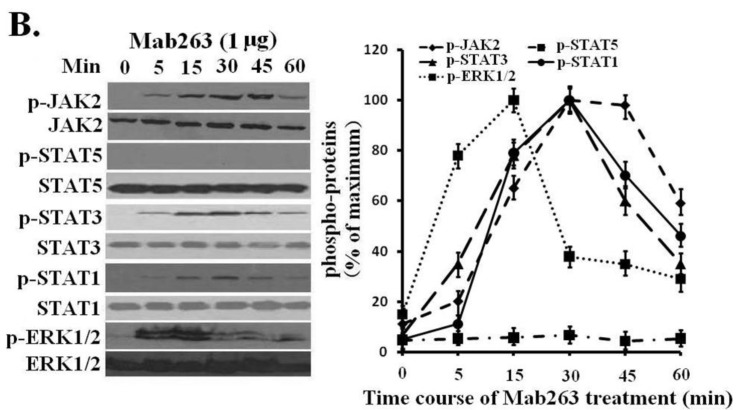
Dose-response and time-course profiles for intracellular signaling proteins activated by Mab263 in the rat GHR cell model. (**A**) Dose response for tyrosine phosphorylation of JAK2, STAT1/3/5 and ERK1/2 induced by Mab263 in CHO-GHR638 cells. The cells were cultured in serum-free medium overnight and incubated with increasing concentrations of Mab263 (0 to 10 μg/mL) for 30 min at 37 °C. After discarding these liquids, the cell layer was quickly washed three times with ice-cold PBS. The cells were then lysed and collected as described in the Materials and Methods. The samples were then subjected to SDS-PAGE, transferred to PVDF membranes, and western blot analysis was performed with the indicated antibodies. (**Right**) The corresponding histogram of data from three separate western blot analyses. Densitometry data for p-JAK2, p-STAT1/3/5 and p-ERK1/2 were normalized to that of total JAK2, total STAT1/3/5 and total ERK1/2. For each signal protein (p-JAK2, p-STAT1/3/5 and p-ERK1/2), the maximum signal achieved in response to Mab263 was considered 100%. The data shown are the mean ± SE; and (**B**) Time course for tyrosine phosphorylation of JAK2, STAT1/3/5 and ERK1/2 induced by Mab263 in CHO-GHR638 cells. The cells were cultured in serum-free medium overnight and incubated with 1 μg of Mab263 for different times (0–60 min) at 37 °C. After removing these liquids, the cell layer was quickly washed three times with ice-cold PBS. The cells were then lysed and collected as described in the Materials and Methods. The samples were then subjected to SDS-PAGE, transferred to PVDF membranes, and western blot analysis was performed with the indicated antibodies. Detection was performed using the ECL detection system. After detection, the membranes were stripped and re-probed with antibodies against the total JAK2, STAT1/3/5 and ERK proteins to verify equal protein loading in each lane. (**Right**) The corresponding histogram of data from three separate western blot analyses. Densitometry data for p-JAK2, p-STAT1/3/5 and p-ERK1/2 were normalized to that of total JAK2, total STAT1/3/5 and total ERK1/2. For each signal protein (p-JAK2, p-STAT1/3/5 and p-ERK1/2), the maximum signal achieved in response to Mab263 was considered 100%. The data shown are the mean ± SE; p, phosphorylated.

Next, dose-response and time-course experiments were conducted in CHO-GHR638 cells and analyzed by western blot. Mab263 phosphorylated JAK2, STAT3, STAT1 (weakly) and ERK1/2 in a time- and dose-dependent manner. Mab263 induced the phosphorylation of JAK2, STAT3 and ERK1/2, and phosphorylation was detectable at 0.1 µg; the phosphorylation levels of JAK2, STAT3 and ERK1/2 were maximized at concentrations of approximately 1–5 µg, and the phosphorylation levels began to decline slightly at higher concentrations (Figure 3A). In the time-course experiments, Mab263-induced tyrosine phosphorylation of JAK2, STATs and ERK1/2 was observed at 5 min, reached the maximum at 15–45 min and, then, declined thereafter (Figure 3B). Because STAT5 phosphorylation cannot be detected using a phospho-specific antibody under our experimental conditions, 4G10, which reacts with the total phosphorylated tyrosine, was chosen to assess the STAT5 and STAT1 phosphorylation again. No tyrosine phosphorylation on STAT5 was detected (data not shown), suggesting that Mab263 does not activate STAT5 and STAT1 under our current experimental conditions.

### 2.3. Signaling Transduction Activated by Mab263 in 3T3-F442A Cells

The above studies were carried out in a rat GHR model. In this section, the mouse GHR model 3T3-F442A, which endogenously expresses abundant GHRs, was selected to further evaluate the intracellular signal transduction induced by Mab263.

**Figure 4:**
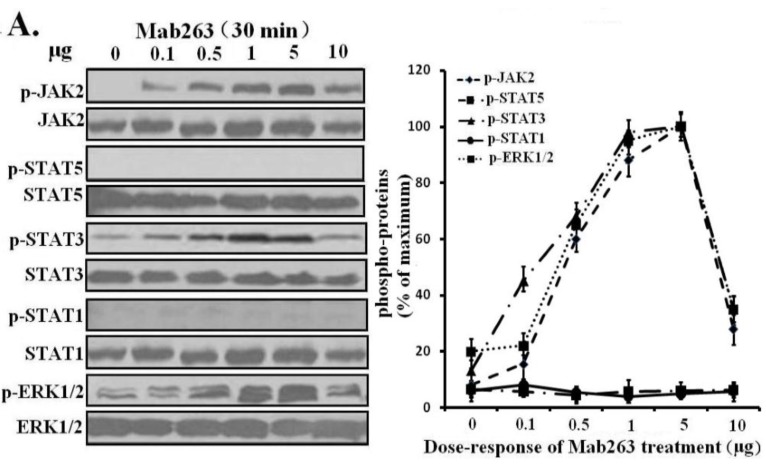

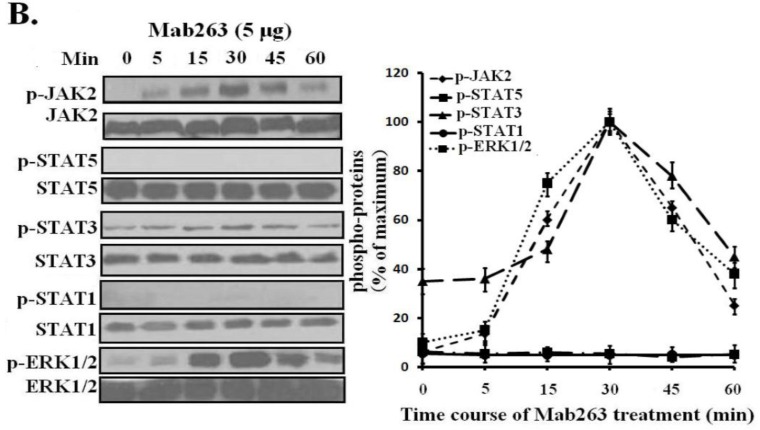
Dose-response and time-course profiles for intracellular signaling proteins activated by Mab263 in the mouse GHR cell model. (**A**) Dose response for tyrosine phosphorylation of JAK2, STAT1/3/5 and ERK1/2 induced by Mab263 in 3T3-F442A cells. The cells were cultured in serum-free medium overnight and incubated with increasing concentrations of Mab263 (0 to 10 μg/mL) for 30 min at 37 °C. After discarding these liquids, the cell layer was quickly washed three times with ice-cold PBS. The cells were then lysed and collected as described in the Materials and Methods. The samples were then subjected to SDS-PAGE, transferred to PVDF membranes, and western blot analysis was performed with the indicated antibodies. (**Right**) The corresponding histograms of the data of three separate western blot analyses. Densitometry data for p-JAK2, p-STAT1/3/5 and p-ERK1/2 were normalized to that of total JAK2, total STAT1/3/5 and total ERK1/2. For each signal protein (p-JAK2, p-STAT1/3/5 and p-ERK1/2), the maximum signal achieved in response to Mab263 was considered 100%. The data shown are the mean ± SE; and (**B**) Time course for tyrosine phosphorylation of JAK2, STAT1/3/5 and ERK1/2 induced by Mab263 in 3T3-F442A cells. The cells were cultured in serum-free medium overnight and incubated with 5 μg of GH for different times (0–60 min) at 37 °C. After removing these liquids, the cell layer was quickly washed three times with ice-cold PBS. The cells were then lysed and collected as described in the Materials and Methods. The samples were then subjected to SDS-PAGE, transferred to PVDF membranes, and western blot analysis was performed with the indicated antibodies. Detection was performed using the ECL detection system. After detection, the membranes were stripped and re-probed with antibodies to the total JAK2, STAT1/3/5 and ERK to verify equal protein loading in each lane. (**Right**) The corresponding histograms of the data of three separate western blot analyses. Densitometry data for p-JAK2, p-STAT1/3/5 and p-ERK1/2 were normalized to that of total JAK2, total STAT1/3/5 and total ERK1/2. For each signal protein (p-JAK2, p-STAT1/3/5 and p-ERK1/2), the maximal signal achieved in response to Mab263 was considered 100%. The data showed are the mean ± SE; p, phosphorylation.

Dose-response and time-course experiments were carried out in 3T3-F442A cells. Mab263 also induced JAK2, STAT3 and ERK1/2 activation in a time- and dose-dependent manner. Mab263 activated JAK2, STAT3 and ERK1/2, and phosphorylation of JAK2, STAT3 and ERK1/2 was observed after stimulation with 0.1 μg Mab263 (Figure 4A). The tyrosine phosphorylation of JAK2, STAT1/3 and ERK1/2 was maximized at 1–5 μg and began to decline thereafter. In the time-course experiments, the phosphorylation of JAK2, STATs and ERK1/2 induced by Mab263 was detected at 5 min, and maximum phosphorylation was observed from 10 to 45 min; the phosphorylation level began to decline thereafter (Figure 4B). Likewise, because STAT5 phosphorylation cannot be detected using a phospho-specific antibody for our experimental conditions, 4G10, which reacts with the total phosphorylated tyrosine, was selected to assess STAT5 and STAT1 phosphorylation again. Tyrosine phosphorylation on STAT5 was not detected (data not shown), suggesting that Mab263 does not activate STAT5 and STAT1 under our experimental conditions.

### 2.4. Signaling Transduction Activated by Mab263 in Vivo

**Figure 5:**
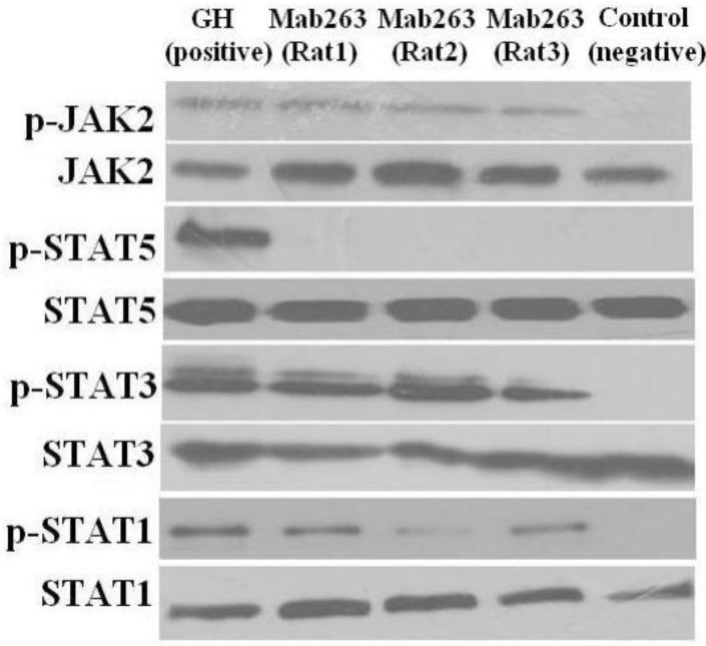

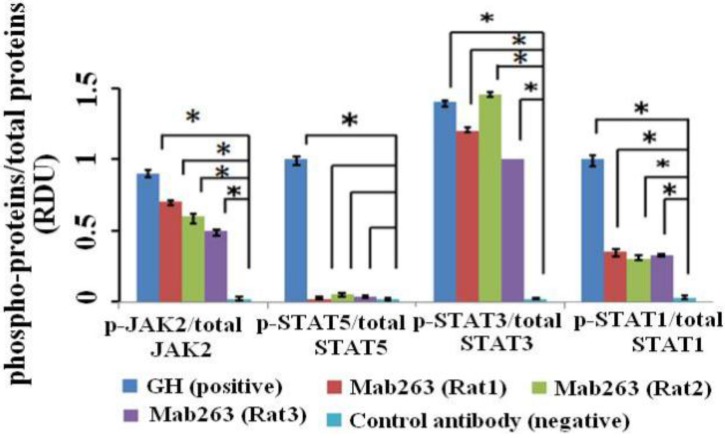
The intracellular signaling pathway(s) induced by Mab263 *in vivo*. Rats were stimulated with GH, Mab263 or control antibody for 20 min, as described in the Materials and Methods. The rats were sacrificed by decapitation, and the livers were quickly removed. The tissue samples were homogenized in ice-cold lyses buffer with a pestle and then maintained at 4 °C for 30 min with continuous shaking. The samples were then centrifuged, collected and lysed directly in SDS-PAGE sample buffer boiled for 5 min and quantitated with a BCA kit. The samples were then separated by SDS-PAGE and transferred to a PVDF membrane. After blocking and washing, the membranes were probed with antibodies recognizing p-JAK2 and p-STAT1/3/5. After washing, secondary antibody (horseradish peroxidase (HRP)-goat anti-mouse) was added to detect the bound antibody using the ECL detection system. After detection, the membranes were stripped and re-probed with antibodies to the total JAK2, STAT1/3/5 and ERK proteins to verify equal protein loading in each lane. (**Bottom**) The corresponding histogram of data from three separate western blot analyses. Densitometry data for p-JAK2, p-STAT1/3/5 and p-ERK1/2 were normalized to that of total JAK2, total STAT1/3/5 and total ERK1/2, presented as RDU and expressed as the means ± SE. * *p* < 0.05 compared with the control values; p, phosphorylation.

*In vivo*, the liver from intact, but not hypophysectomized, rats has been used as a model to investigate intracellular signaling triggered by GH [20,21]. Therefore, we also analyzed the intracellular signaling induced by Mab263 *in vivo*. Sprague-Dawley (SD) rats were treated as described in the Materials and Methods. The representative results are shown in Figure 5. Phosphorylated proteins, including JAK2, STAT3 and STAT1, but not STAT5, could be easily detected with GH stimulation, and Mab263 stimulation also phosphorylated JAK2 and STAT3, weakly phosphorylated STAT1, but did not phosphorylate STAT5; the control antibody showed no effect. These results suggested that Mab263 also triggered intracellular signaling pathways (JAK2, STAT1/3, but not STAT5) *in vivo*, and the differences between the control antibody and the Mab263 treatments were statistically significant (*p* < 0.05).

### 2.5. Discussion

As a well-known GHR agonist, Mab263 has been extensively studied for its agonist property *in vitro* and *in vivo* [5,6,7,8,9,10]. However, the intracellular signaling pathway activated by Mab263 has remained unclear. Therefore, we focused our current work on intracellular signaling pathways triggered by this Mab *in vivo* and *in vitro* and found that Mab263 activated different signaling transduction pathways than GH. These findings suggest that Mab263 has unique biological functions and roles, which imply that Mab263 possesses important potential applications: (1) Mab263 may serve as a signal-specific GHR agonist. Because it can activate different signaling pathways, it may exhibit the different physiological functions compared with GH; (2) Mab263 may be used as a useful tool to investigate the relationship between GHR conformation change(s) and GHR-mediated intracellular signaling, which is a current hot topic in GH-related studies; and (3) This study may imply a strategy for designing signal-specific cytokine agonists using anti-GHR antibodies (such as Mab263). In addition, more biologic properties and potential applications may be found with the in-depth study of Mab263.

A GH-induced GHR conformational change has been generally accepted to be required for its activation, although the nature of this conformational change remains unclear. Recently, Waters and colleagues proposed a new model in which the GHR exists predominantly as a dimer held together by its transmembrane helices, and GH binding to GHR rotates the subunits and subsequently converts the transmembrane helices into a left-hand crossover state, which repositions the intracellular domains (ICDs) and activates JAK2 [22,23]. Similarly, the computational studies of Poger and Mark [24] and Pang and Zhou *et al*. [25] also supported this view. We analyzed the possible reasons why Mab263 exhibits signal-specific agonist activity based on the above-motioned mechanism of GHR activation. Briefly: (1) Based on the Mab263 binding property, GH is an asymmetric molecule that initiates its interaction via two non-equivalent binding sites with GHR. In contrast, Mab263 (IgG) is a symmetric molecule that contains two identical binding sites with the same binding affinity. Thus, Mab263 should simultaneously bind the two moieties of the receptor dimer. Therefore, the GHR conformation change(s) induced by Mab263 could not be different compared with GH, which causes Mab263 to trigger different intracellular signaling pathways; (2) Based on the structural characteristics of Mab263 (IgG), the crystallographic study showed that a typical IgG-type antibody molecule possesses a rigid tripod-like shape. In contrast, the GH molecule shows a flexible structure by crystallographic study [26]. It has been known that the torsional force of the ligand is required for GHR conformation change(s) and GHR activation, and the flexible molecules (such as GH) are more suitable for generating the torsional force compared with the rigid molecules (such as Mab263), which may also result in the GHR conformation change(s) induced by Mab263 not being different compared with GH; and (3) Wu *et al*. [9] used a modelling analysis to report that Mab263 can induce similar, but not identical, conformational changes compared with GH.

The relationship between JAK2 and downstream signaling molecules (such as STAT1/3/5, ERK) is not fully understood. Only a few studies have examined this relationship. JAK2 is known to directly phosphorylate STAT1/3, which avoids the direct interaction between GHR and STAT1/3 [27]. In contrast to STAT1/3, active JAK2 activated the tyrosine residues in the carboxyl terminal domain of the GHR, which, in turn, results in phosphorylation of STAT5 [28]. Mab263 activated JAK2 in the current study, which agrees with Wan’s modelling analysis [9]; interestingly, STAT5 phosphorylation was not observed in the rat GHR model, and STAT5/1 activation was not observed in the mouse GHR model under our current experimental conditions. We speculated that Mab263 induces a similar, but not identical, conformational change, as mentioned above, which induces different signaling pathways. In addition, some similar observations have been documented; a study by Rowlinson and colleagues [29] indicated that an agonist-induced conformational change in the GHR determines the choice of signaling pathway.

Although a series of studies have reported that Mab263 exhibits agonist-like properties, it has also been shown to act as a weak agonist compared to the application of GH *in vitro* or *in vivo* [5,6,7,8,9]. To date, the reason for this weak agonist activity compared to GH remains unclear. Our current study provides a possible explanation. The JAK2-STAT5 signaling pathway is a key mediator of GH-induced physiological functions; and GH-induced *insulin-like growth factor-1* (*IGF-1*) gene expression is mostly mediated by STAT5 activation. However, we found that Mab263 cannot activate STAT5 *in vivo* and *in vitro* in this study (nevertheless, the active level of STAT5 may have been too weak to be detected by western blotting under our experiment conditions), which may be the reason for the partial function of Mab263 compared to GH.

Primate GHs (humans and monkeys) can bind to and activate non-primate GHRs, as well as primate GHRs, whereas the GHs from non-primates are ineffective in primates [30,31]. Nevertheless, Mab263, which was produced from mice by immunization with a human GH affinity-purified preparation of the rabbit or rat liver GH receptor, is a broad-spectrum agonist that can activate human GHR, rat GHR and mouse GHR [9]. Furthermore, we speculated that it could also activate the GHR of otherspecies (such as pig and bovine GHR, *etc*.) based on the epitope it recognizes, and it could also exhibit a different intracellular signaling property on the GHR of other species. Of course, this is only speculation, and further investigation is necessary to elucidate this question. 

Collectively, our results suggest that: (1) Mab263 is not a suitable GH mimic due to its biased signaling property; (2) Mab263 may serve as a signal-specific agonist; (3) Mab263 may serve as a valuable biological reagent to investigate the relationship between GHR conformation change(s) and GHR-mediated intracellular signaling pathways, which is a current hot topic; and (4) this study may imply a strategy for designing signal-specific cytokine agonists using anti-GHR antibodies (such as Mab263).

## 3. Experimental Section

### 3.1. Antibodies and Reagents

Mab263 is a mouse monoclonal antibody raised against purified rabbit or rat GHR, which recognizes a variety of GHRs, including human, rabbit and rat receptors [4,9]. Mab263 was purchased from Abcam (Cambridge, UK). Rabbit monoclonal antibodies for total JAK2 and p-JAK2 (Tyr1007/1008), total STAT5 and p-STAT5 (Tyr694), total STAT3 and p-STAT3 (Tyr705), total STAT1 and p-STAT1 (Tyr701) and total ERK1/2 and p-ERK1/2 (Thr202/Tyr204) were purchased from Cell Signaling Technology (Danvers, MA, USA); these antibodies cross-react with human, mouse and rats epitopes. The anti-rabbit IgG horseradish peroxidase conjugate was obtained from Sigma (St. Louis, MO, USA). Human growth hormone (hGH) was purchased from Sigma (St. Louis, MO, USA). hGH conjugated with FITC was prepared in our lab [11]. Bovine serum and Dulbecco’s Minimal Eagle’s Medium with high glucose (hDMEM) were purchased from Gibco (Grand Island, NY, USA). Bovine serum albumin (BSA), cell lysis buffer (RIPA kit), enhanced chemiluminescence (ECL), Difco skim milk and BCA kits were obtained from Pierce (Rockford, IL, USA). Polyvinylidene fluoride (PVDF) membranes were obtained from Millipore (Bedford, MA, USA). Unless otherwise stated, all other reagents were from Sigma (St. Louis, MO, USA).

### 3.2. Cell Line and Cell Culture

CHO cells stably transfected with the full-length rat GHR (referred to as CHO-GHR638) were prepared in our lab [25] and maintained in 5% CO_2_ at a relative humidity of 95% and 37 °C in Ham’s F-12 nutrient mixture supplemented with 10% fetal calf serum (FCS), 100 U/mL penicillin and 100 μg/mL streptomycin. In addition, a Scatchard analysis determined that each CHO cell used in this study expresses 13,200 ± 450 receptors [25]. The 3T3-F442A mouse pre-adipocyte cell line, which was initially developed by Green [12], was provided by Wei Xing (Hua Cheng Biotechnology Co., Ltd., Changchun, China). The 3T3-F442A cells were cultured in hDMEM containing 10% bovine serum, 2% antibiotics (100 U/mL of penicillin and 100 mg/mL of streptomycin), 2.5 mM glutamine (standard medium) and 0.3 mg/mL fungizone, grown in six-well plastic tissue culture plates in a humidiﬁed incubator containing an atmosphere of 5% CO_2_/95% air at 37 °C. The cellular morphology was observed with an inverted phase contrast microscope (CK×41; OlympusCK-41, Tokyo, Japan).

### 3.3. Receptor Binding Analysis

The ability of Mab263 to bind 3T3-F442A cells under our experimental conditions was assessed using flow cytometric analysis. 3T3-F442A cells were digested and detached from the tissue culture dishes with trypsin, and the cells were then washed with PBS and resuspended in 0.5 mL FACS buffer. FITC-GH (as a positive control), FITC-Mab263 or control antibody (as a negative control) was added to the FACS buffer, and the mixture was incubated for 1 h in the dark at 4 °C. The cells were then washed with PBS, resuspended in 0.5 mL FACS buffer and analyzed using a FACS Calibur Flow Cytometer (Becton Dickenson, San Jose, CA, USA). The data were analyzed using the Cell Quest software (Becton Dickenson, San Jose, CA, USA).

Next, a competitive receptor binding analysis was performed to further confirm the ability of Mab263 to bind to the mGHR expressed on 3T3-F442A. 3T3-F442A cells were incubated in serum-free medium containing 1% BSA for 10 h. The cells were then detached and collected by centrifugation. After washing three times with PBS, the cells were resuspended in binding buffer (serum-free media containing 1 mg/mL BSA), pipetted into FACS tubes (1 × 10^6^ cells/tube) and then incubated with FITC-GH (15 nM) or increasing concentrations of unlabeled GH, Mab263 or control antibody for 1 h in the dark at 4 °C. After the incubation, the cells were washed three times, resuspended in 0.5 mL FACS buffer and analyzed using on a FACS Calibur Flow Cytometer. The data were analyzed using the Cell Quest software.

### 3.4. Analysis of Intercellular Signaling by Western Blot in the rGHR or mGHR Cell Model

The CHO-GHR638 or 3T3-F442A cells were grown in 6-well plates and cultured as described above. Before the experimental incubations, the medium of the CHO-GHR638 or 3T3-F442A cells was replaced with fresh serum-free medium containing 1% BSA, and the incubation was continued for 10 h at 37 °C. For the dose-dependent experiments, the cells were treated with the indicated concentration of Mab263, GH or control antibody for different times. For the time-course experiment, the cells were treated with the indicated concentration of Mab263, GH or control antibody for different times. All incubations were carried out on a shaking table at a constant temperature of 37 °C. At the end of the treatment, the medium was discarded, and the cells were washed three times with ice-cold PBS. The total protein was obtained by adding 300 μL of lysis buffer (1% (*v/v*) Triton X-100, 150 mM NaCl, 10% (*v/v*) glycerol, 50 mM Tris-HCl (pH 8.0), 100 mM of NaF, 2 mM EDTA, 1 mM phenylmethylsulfonyl fluoride, 1 mM sodium orthovanadate, 10 mM benzamidine, 5 μg/mL aprotinin and 5 μg/mL leupeptin) to the cells in each well on ice at 4 °C for 30 min, scraping the dissolved cells with a cell scraper, collecting the mixture and subjecting the mixture to ultrasonication 3 times. The supernatant was collected by centrifugation (12,000× *g*, 10 min) at 4 °C. The samples were directly lysed in SDS-PAGE sample buffer, boiled for 5 min and quantitated with a BCA Protein Assay Reagent Kit (Beyotime institute of Biotechnology, Haimen, China) according to the manufacturer’s instructions. The samples (50 μg of protein per lane) were separated with SDS-PAGE using 10% polyacrylamide gels and transferred to PVDF membranes. The membranes were washed three times for 10 min each and blocked with 5% non-fat milk for 2 h at 37 °C After three washes with TBST, the membranes were incubated with primary antibodies (p-JAK2, p-STAT1/3/5 and p-ERK1/2) according to the manufacturer’s protocols, and the membranes were then washed three times with TBST and incubated with HRP-conjugated goat anti-rabbit IgG antibody (secondary antibody) for 1 h at 37 °C. The proteins were subsequently detected with the ECL detection system. After ECL detection, the membranes were stripped of the primary and secondary antibodies according to the manufacturer’s instructions. The stripped membranes were blocked and re-probed for JAK2, STAT1/3/5 or ERK1/2. Densitometric analysis of the immunoreactive protein bands was performed using Quantity One software (Bio-Rad, Hercules, CA, USA).

### 3.5. Analysis of Intracellular Signaling by Western Blot in Vivo

Eighteen male SD rats (Animal Lab of Jilin University), six-week-old and weighing 180–200 g, were housed in three individuals per cage in the conventional animal room with free access to standard rodent chow and water. After a 3 days adaptation period, the rats were anesthetized. The abdominal cavity was opened, the portal vein was exposed and GH, Mab263 or control antibody was injected at a dose of 1.5 mg/kg body weight (GH) or 5 mg/kg body weight (Mab263 or control antibody) in a volume of 1 mL of physiological saline. Twenty minutes after the injection (when the liver sensitivity to GH is maximized based on our personal observation), the rats were sacrificed by decapitation and the livers were quickly removed and frozen in liquid nitrogen until analysis. The study protocol was approved by the Animal Ethical Committee of Jilin Agricultural University.

The liver samples were homogenized in 5 volumes (*w/v*) of ice-cold lysis buffer with a pestle and then incubated at 4 °C for 30 min with continuous shaking. The samples were then centrifuged for 10 min at 12,000× *g* at 4 °C, and the supernatant was collected. The samples were lysed directly in SDS-PAGE sample buffer, boiled for 5 min and quantitated with the BCA Protein Assay Reagent Kit according to the manufacturer’s instructions. The samples were separated by SDS-PAGE and transferred to PVDF membranes, followed by western blot analysis, as described in Section 3.4. Densitometric analysis of the immunoreactive protein bands was performed using Quantity One software.

### 3.6. Statistical Analysis

The data collected from three independent experiments are presented as the mean values ± standard error (SE). Statistical analysis was done by one-way analysis of variance (ANOVA) using Statistical Analysis System (SAS) software (SAS version 9.0; Institute Inc., Cary, NC, USA), followed by the Student-Newman-Keuls test. *p* < 0.05 was considered statistically significant.

## 4. Conclusions

In this study, we focused our current work on intracellular signaling pathways triggered by Mab263 *in vivo* and *in vitro* and found that it activated different signaling transduction pathways than GH. These findings indicate that: (1) Mab263 may serve as a signal-specific GHR agonist; (2) Mab263 may serve as a valuable biological reagent to investigate the relationship between GHR conformation change(s) and GHR-mediated intracellular signaling pathways; and (3) this study may imply a strategy for designing signal-specific cytokine agonists using anti-GHR antibodies (such as Mab263).

## Abbreviations

hGH: human growth hormone
GHR: growth hormone receptor
Mab: monoclonal antibody
FITC: fluorescein isothiocyanate
PBS: phosphate-buffered saline
JAK: Janus kinase
STAT: signal transducers and activators of transcription
ERK: extracellular regulated protein kinases
